# Supplementary material for: Integrated Analysis of the Transcriptome and Metabolome of Brassica rapa Revealed Regulatory Mechanism under Heat Stress
Source: Int J Mol Sci. 2023 Sep 12;24(18):13993. doi: 10.3390/ijms241813993 (PMC10531312; doi:10.3390/ijms241813993)
Supplement: Supplementary file 1 [file ijms-24-13993-s001.zip › ijms-2583938-supplementary.pdf]

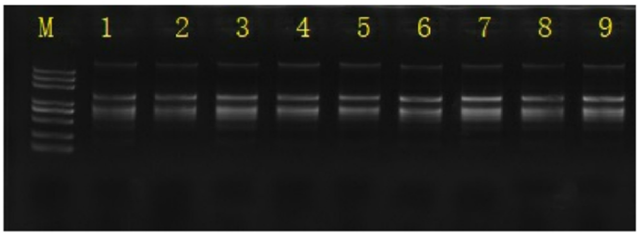

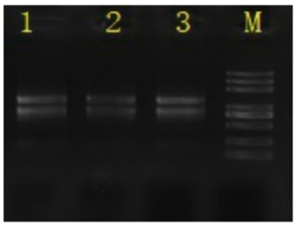

Figure S1 Gel electrophoregram for RNA

Table S1 The concentration and OD260/280 ratio of RNA used for transcriptome sequencing

Sample ID	concentration（ng/nl）	OD260/280 value
AJH1_1	697.7	2.03
AJH1_2	645.4	2.01
AJH1_3	632.1	2.06
AJH2_1	724.4	1.98
AJH2_2	794.3	2.05
AJH2_3	608.8	2.03
WYM1_1	627.9	1.97
WYM1_2	680.0	1.95
WYM1_3	632.7	2.05
WYM2_1	610.1	2.07
WYM2_2	647.4	2.07
WYM2_3	647.4	1.98

Table S2 The Specific primers sequences used for qRT-PCR

Gene ID	forward primer	reverse primer
BraA06g001970.3C	TTCAAGGAGTTATGGTTA	TAGGAATCACAACATTAAG
BraA08g014770.3C	AATGTGTTCAATCTTCTC	GAATATACTGTAGGTTGTTAT
BraA03g027160.3C	GATGAAGTGAAGATAGAG	TATCAACATTATCAGGTAG
BraA03g009000.3C	ATCATCAACACCTTCTAC	ATACAACAACAACAGTAATC
BraA04g010550.3C	TTCTTAACCTAAACAACATTCCAT	CTTCCTTCTTCAGTCCTTCT
BraA03g011180.3C	ACAAGAACATACTTCAGAT	CAACTTAAACCTCCTCAT
BraA03g005120.3C	TGTGAAGTATGTGAGGAT	GTTACAAACAGCAGAGAT
BraA10g018140.3C	ATAAGAAGGATGTTAGTG	AAGAATCAATCTCAATAGT

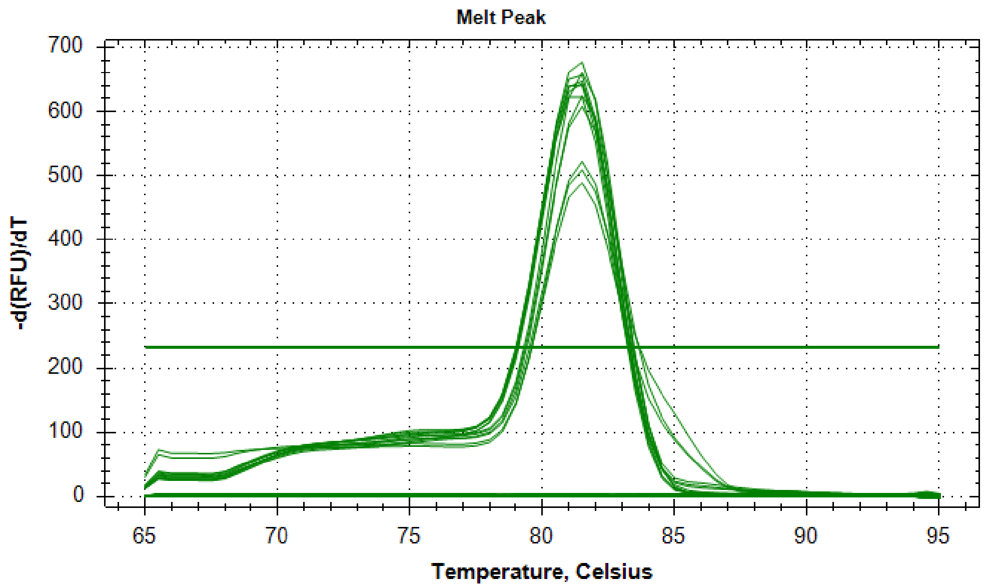

Figure S2 The melting curve for qRT-PCR.
